# 2031. Role of Urine Culture Hard Stop in Reducing Catheter-associated Urinary Tract Infections

**DOI:** 10.1093/ofid/ofac492.1654

**Published:** 2022-12-15

**Authors:** John Cherian, Robert Tibbetts, Paula Robinson, Tarlisha Holsey, George J Alangaden, Geehan Suleyman

**Affiliations:** Henry Ford Health, Detroit, Michigan; Henry Ford Health, Detroit, Michigan; Henry Ford Health, Detroit, Michigan; Henry Ford Health, Detroit, Michigan; Henry Ford Health, Detroit, Michigan; Henry Ford Health, Detroit, Michigan

## Abstract

**Background:**

Catheter-associated urinary tract infection (CAUTI) is one of the most commonly reported healthcare associated infections (HAI) reported to the National Healthcare Safety Network (NHSN). However, asymptomatic bacteriuria is common in patients with indwelling urinary catheters (IUC), and inappropriate culturing leads to overdiagnosis in colonized patients, unnecessary antibiotics and increased resistance. We evaluated the effectiveness of an electronic medical record (EMR) “hard stop” in reducing inappropriate urine cultures (UC) and its impact on CAUTI rates.

**Methods:**

This was a pre-post quasi-experimental retrospective study comparing CAUTI rate per 1000 catheter days, UC order rate per 1000 patient days, Standardized Utilization Ratio (SUR) and Standardized Infection Ratio (SIR) in the pre-intervention period (January 2019-December 2020) to the intervention period (April 2021-March 2022) in Southeast Michigan. In March 2021, we implemented a hard stop in Epic® that fired 24 hours after admission in patients with IUC >1 calendar day and until 4 days after IUC removal. The Medical Director of Infection Prevention and Control had the ability to override the hard stop when indicated after reviewing the case upon provider request; education was provided in real-time. We prospectively monitored outcomes, including pyelonephritis and/or bacteremia within 3 days and readmission for sepsis within 30 days in patients UC was deemed unnecessary.

**Results:**

CAUTI rate was 0.52 in the pre-intervention period and 0.09 in the post-intervention period, an 83% reduction (Figure 1). Rate of UC performed was 0.283 in the pre-intervention period and 0.218 in the post-intervention period, a 23% reduction. SUR decreased from 0.809 to 0.716, an 11% reduction (95% CI, 0.875-0.894, p< 0.001). SIR decreased from 0.392 to 0.135, a 66% reduction (95% CI, 0.154-0.0689, p=0.0015). Post-intervention, there were 44 override requests that were denied with no adverse patient outcomes.

**Conclusion:**

Urine culture stewardship, utilizing an electronic hard stop, was effective in reducing inappropriate UC orders, SIR and SUR in patients with IUC without causing patient harm. This strategy combined with real-time education can significantly reduce CAUTI rates.

**Figure:**
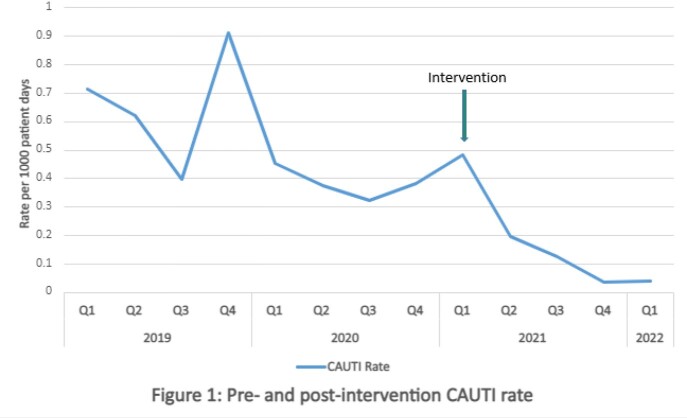

**Disclosures:**

**All Authors**: No reported disclosures.

